# Poorer mental well-being and prior unmet need for mental healthcare: a longitudinal population-based study on men in Sweden

**DOI:** 10.1186/s13690-021-00706-0

**Published:** 2021-11-03

**Authors:** Sara Olsson, Bo Burström, Gunnel Hensing, Jesper Löve

**Affiliations:** 1 School of Public Health and Community Medicine, Institute of Medicine, University of Gothenburg, Box 453, 405 30 Gothenburg, Sweden; 2 Department of Global Public Health, Karolinska Institutet, 171 77 Stockholm, Sweden

**Keywords:** Longitudinal studies, Mental health services, Unmet need, Barriers to care, Health behaviours, Patient satisfaction, Mental disorders, Depression, Gender, Masculinity

## Abstract

**Background:**

Depression and anxiety disorder contribute to a significant part of the disease burden among men, yet many men refrain from seeking care or receive insufficient care when they do seek it. This is plausibly associated with poorer mental well-being, but there is a lack of population-based research. This study investigated 1) if men who had refrained from seeking mental healthcare at any time in life had poorer mental well-being than those who sought care, 2) if those who had sought care but perceived it as insufficient had poorer mental well-being than those who had perceived care as sufficient, and 3) if these differences persisted after 1 year.

**Methods:**

This longitudinal study used questionnaire data from a population-based sample of 1240 men, aged 19–64 years, in Sweden. Having refrained from seeking mental healthcare, or perceiving the care as insufficient, at *any time in life*, was assessed in a questionnaire, 2008. Current mental well-being was assessed in 2008 and 2009 using mean scores on the WHO (Ten) Well-being Index. Lower scores indicate poorer mental well-being. Group differences were calculated using t-tests and multivariable linear regression analysis.

**Results:**

Of the men who had perceived a need for mental healthcare, 37% had refrained from seeking such care. They had lower mental well-being scores in 2008, compared to those who sought care. Of those seeking care, 29% had perceived it as insufficient. They had lower mental well-being scores in 2008, compared to those who perceived the care as sufficient, but this was not statistically significant when controlling for potential confounders. There were no differences in mental well-being scores based on care-seeking or perceived care-sufficiency in 2009.

**Conclusions:**

This population-based study indicates that men who have previously refrained from seeking mental healthcare, or perceived the care as insufficient, have poorer mental well-being. However, the lack of differences at the one-year follow-up contradicts these results. The results highlight the need for larger longitudinal studies, measuring care-seeking within a more specified time frame. This should be combined with efforts to increase men’s mental healthcare-seeking and to provide mental healthcare that is perceived as sufficient.

**Supplementary Information:**

The online version contains supplementary material available at 10.1186/s13690-021-00706-0.

## Introduction

Common mental disorders such as depression and anxiety disorders have a high prevalence [1], and account for 4% of the disease burden among men aged 15–49 years in Western Europe, according to the Global Burden of Disease Study [2]. Especially if untreated, these disorders are often longstanding, recurrent, and have a detrimental effect on individual function and productivity [3–5]. There is consistent evidence for the benefit of receiving treatment, even for mild to moderate depression and anxiety disorders [4, 5]. Remission or reduction of symptoms can be attained by psychotherapy, pharmacotherapy, or a combination of both [4, 5]. Yet a large proportion of men refrain from seeking mental healthcare. For example, a population-based study from Sweden showed that 57% of the men with current depression and/or anxiety disorder had not sought care during the past year [6].

Refraining from seeking mental healthcare has been suggested to be detrimental for men’s mental well-being on a population level [7–11]. Yet empirical data is lacking. For example, men’s reluctance to seek care has been hypothesised to be one of the explanations for men’s higher risk of premature death and suicide [7]. This is partly due to an assumed negative effect of later diagnosis and treatment [8, 9, 12] and a proposed connection to other risk behaviours e.g. higher alcohol consumption [7], reckless driving [10], and workaholism [9]. These behaviours have partly been explained by masculinity norms encouraging men to deal with mental health problems in ways that are harmful to their health, but which may benefit men’s position in social hierarchies [7]. Some empirical research indicates detrimental outcomes of refraining from seeking mental healthcare among men, but these do not focus specifically on mental healthcare-seeking; rather, they have a broader perspective on masculinity norms and suicide [13, 14]. For example, a population-based study identified self-reliance, defined as being reluctant to seek help, as a risk factor for suicidal thinking among men [13]. A qualitative study on men who had attempted suicide found that due to masculinity norms that encouraged non-disclosure of distress these men had opted for suicide instead of seeking care [14]. Although most men who refrain from seeking mental healthcare do not deteriorate into suicidality due to having milder conditions [15, 16] they may still benefit from treatment [4, 5].

Based on the suggested risks for poorer health due to lack or delay of treatment, and risk behaviours among men who have refrained from seeking mental healthcare [7–12], one might assume that these men will have poorer subsequent mental well-being than those who seek care. However, there is a lack of evidence from population-based longitudinal studies to confirm this hypothesis.

Furthermore, a limitation of previous research is that most studies have focused on men’s reluctance to seek mental healthcare [17], overlooking the fact that many men do seek it. However, many care-seeking men receive insufficient care. Insufficient care can be defined using both patient perceptions, e.g. perceived unmet need for care [18, 19], and using clinical measures, e.g. inadequate standard of care in regard to evidence-based guidelines [20]. A multi-country study from high-income countries shows that among men and women who sought mental healthcare with symptoms corresponding to major depression, only half received treatment meeting minimally adequate standards [20]. Qualitative studies have also shown that many care-seeking men are sceptical about treatment, minimise their symptoms, and are unwilling to disclose distress, behaviours shown to be related to masculinity norms [14, 17, 21]. Clinical and epidemiological studies have found that men have a higher likelihood for under-diagnosis of depression [22, 23], under-treatment with antidepressants, [24], insufficient follow-up during sick leave for mental diagnoses [25], and of perceiving the mental healthcare as insufficient, than do women [26]. Possibly, this indicates poorer standard and quality of mental healthcare for men than women. Indicators of poor quality of care, such as poor adherence to treatment, and adverse events in care situations, are consistently associated with perceived insufficient care [27]. Based on the association between quality of care and perceived sufficiency of care [27], and the importance of receiving treatment [4, 5], one might assume that men who have sought mental healthcare but perceived it as insufficient, would have poorer subsequent mental well-being than those who perceived the care as sufficient. To our knowledge, no present study investigates this hypothesis using longitudinal data from a population-based sample of men.

Considering that men’s unmet need for mental healthcare occurs on multiple steps on the pathway to mental healthcare [26], there is a need for epidemiological studies considering how both *refraining from seeking care*, and *perceiving the care as insufficient when seeking it*, are related to subsequent poorer mental well-being. There is a particular need for longitudinal studies measuring mental well-being at multiple time points. Firstly, a persistent difference in mental well-being based on prior unmet need implies a more stable effect than a difference observed at one-time point only. Secondly, poorer mental well-being based on prior unmet need may persist over time due to the longstanding and recurrent nature of untreated depression and anxiety disorders [3, 4, 28]. Thirdly, men who have postponed care-seeking, or received insufficient care when seeking it, may have poorer treatment outcomes [8, 9, 12, 29]. Fourthly, they may engage in risk-behaviours that put an additional burden on their mental well-being [7, 9, 10]. Longitudinal population-based studies on men’s mental healthcare-seeking and subsequent mental well-being are important to estimate the significance and societal burden of men’s unmet need for mental healthcare. As research on men’s health is a neglected area, more research is needed to guide policy and interventions and put men’s unmet need for mental healthcare on the agenda [30]. Very few longitudinal studies are conducted [21], and research from Sweden is especially valuable considering that previous research on men’s mental healthcare-seeking is primarily from the US, Australia, and the UK, with few studies from the Nordic countries [31].

**Fig. 1:**
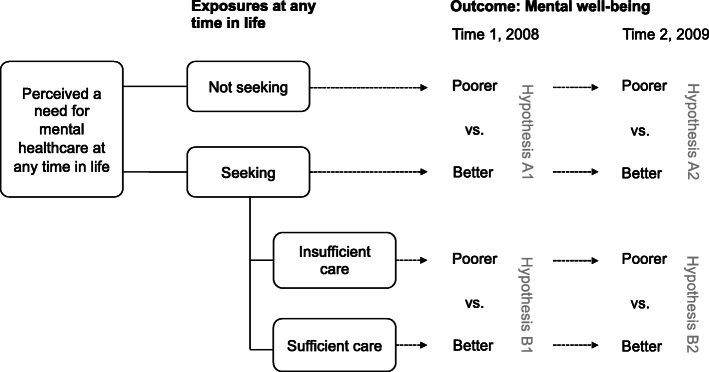
Hypotheses and study design

In a population-based sample of men in Sweden, this longitudinal study investigated 1) if men who had *refrained from seeking mental healthcare* at any time in life had poorer mental well-being than those who sought care, 2) if those who had sought care but *perceived it as insufficient* had poorer mental well-being than those who perceived care as sufficient, and 3) if these differences persisted after 1 year. As the measurement of *refraining from seeking mental healthcare* and *perceiving it as insufficient* referred to “any time in life”, the outcome, i.e., poorer mental well-being, could have occurred already at baseline, time 1. Therefore, mental well-being was measured at two time points, 1 year apart, time 1 (T1) and time 2 (T2). The two measurement points gave the opportunity to investigate whether potential differences were stable over time. Thereby, this design allowed for challenging results provided by a cross-sectional design only. The study design and hypotheses are illustrated in Fig. 1. The following hypotheses were tested:

A. Men who have refrained from seeking mental healthcare at any time in life will have poorer mental well-being at both T1 (hypothesis A1) and T2 (hypothesis A2), compared to men who sought care when perceiving a need.
B. Men who have sought mental healthcare at any time in life, but perceived the care as insufficient, will have poorer mental well-being at both T1 (hypothesis B1) and T2 (hypothesis B2), compared to those who perceived it as sufficient.

## Methods

### Study design and participants

**Fig. 2:**
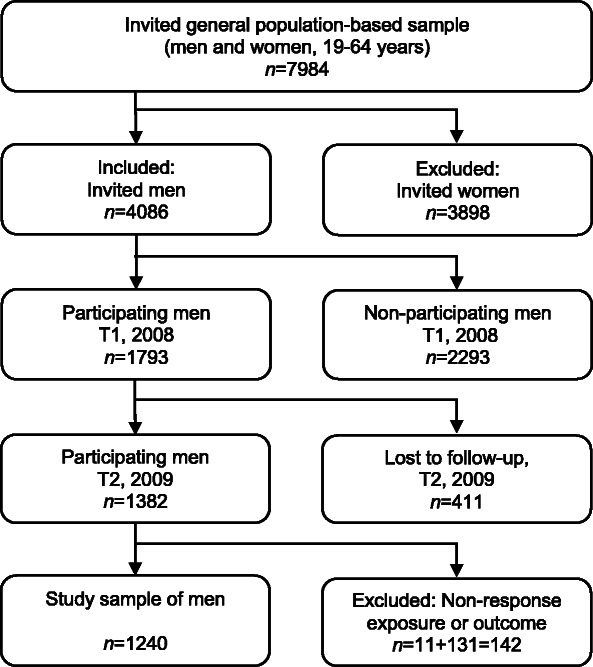
Flow chart of the study sample of men

This longitudinal study was based on secondary analysis of data collected for the purpose of investigating mental health and sickness-absence, the Health Assets Project (HAP) [32, 33]. From HAP, we used two questionnaires and sociodemographic registry data from a general population-based sample of men in Region Västra Götaland, Sweden. The region has 1.7 million urban and rural inhabitants, constituting 17% of Sweden’s population. A random general population-based sample of men and women, aged 19–64 years, was extracted by Statistics Sweden and invited to participate (*n* = 7984, Fig. 2). The invited men and women received the first postal questionnaire, referred to as T1, between 15 April to 30 June 2008 [33]. The T1 questionnaire comprised questions on mental healthcare-seeking at any time in life, mental and physical persistent illness, sociodemographic factors, and an index on mental well-being. A previous analysis of non-participation showed that those born outside the Nordic countries, those with low income, young persons, men, and those who were single were less likely to participate at T1 [34]. Previously, several studies have been published based on the T1 questionnaire and the population-based sample, e.g., a recently published study showing gender and education-based differences in unmet need for mental healthcare [26]. The current study included men only.

Of the 4086 persons registered as men by Statistics Sweden, 44% (*n* = 1793) participated at T1 and consequently were included in this study (Fig. 2). This group received a follow-up questionnaire between 21 September and 12 December 2009, referred to as T2. Both questionnaires were followed by two reminders. The T2 questionnaire comprised an index on mental well-being. Of the 1793 men who participated at T1, 23% were lost to follow-up at T2. In total, 34% of the invited men participated at both T1 and T2 (*n* = 1283, Fig. 2). Of those, 11 participants were excluded due to missing data on mental healthcare-seeking. Also, 131 participants were excluded due to missing data on the item on mental well-being at T1 and/or T2 (*n =* 74 had missing data at T1, *n* = 66 at T2, and *n* = 9 at both T1 and T2). Consequently, the final study sample comprised 1240 men, i.e. participants with data from both T1 and T2 (Fig. 2). The time elapsed between T1 and T2 ranged from 14.5 to 20 months. In this study, the exposures were measured at T1 but referred to “any time in life”. The outcome mental well-being was measured at both T1 and T2, referring to the previous week (Fig. 1).

### Exposure variables

Having perceived a need for mental healthcare and having sought mental healthcare at *any time in life* was assessed using the T1 questionnaire, based on the question “Have you at any time felt so mentally unwell that you felt a need to seek care?” The study sample was divided into three categories based on the response alternatives (“yes”, “yes, but did not seek”, and “no”): 1) *care-seekers*, who had perceived a need for mental healthcare and sought care, 2) *non-care-seekers*, who had perceived a need for mental healthcare but refrained from seeking it, and 3) *non-need-perceivers*, who had not perceived a need for mental healthcare. In addition, care-seekers were divided into two categories based on the follow-up question “Do you think you received the care that you needed?” with the response alternatives “yes” (i.e. *sufficient care-perceivers*) and “no” (i.e. *insufficient care-perceivers,* Fig. 1). Data on reasons for refraining from seeking mental healthcare and where care-seekers had sought care is presented elsewhere [26].

### Potential confounders

The association between poorer mental well-being and refraining from seeking mental healthcare or perceiving the care as insufficient may be confounded by sociodemographic and health variables. For example, lower education is associated with both refraining from seeking mental healthcare [26], and poorer mental well-being [35]. Poor health, e.g. having a persistent mental illness is associated with seeking mental healthcare [36], perceiving the care as insufficient [37, 38], and poorer mental well-being [39–41]. Based on previous research and our analyses of potential associations using directed acyclic graphs (see Additional file 1), potential confounders chosen were level of education, country of birth, age, persistent physical illness, and persistent mental illness. These variables were measured at T1. Age (categorised into 19–30, 31–50, and 51–64 years) and country of birth (dichotomised into Nordic versus non-Nordic country based on nine categories: Sweden, other Nordic countries, other European countries, Africa, Asia, North America, South America, Oceania, and others) were based on register data from Statistics Sweden. The level of completed education was based on questionnaire data (categorised into primary education or less, secondary education, and university education, based on six response alternatives). Persistent illness was measured using questionnaire data on whether the respondent had any persistent disease, illness, or disability, followed by a checklist of categories (e.g., cardiovascular, neurological, and mental illnesses). Respondents choosing “mental illness” were considered to have persistent mental illness. Those choosing one or more physical illnesses categories were considered to have persistent physical illness.

### Outcomes

Mental well-being was assessed both at T1 and T2 using the WHO (Ten) Well-being Index (WHO-10) [42]. The index comprises ten items covering depression, anxiety, energy, and positive well-being in the previous week. Each item has four response alternatives ranging from “never” (i.e. 0) to “all the time”, (i.e. 3), giving a total score of 0–30. A lower score indicates lower mental well-being and has been found to correspond to depression [39–41], and suicidality [41]. For example, cut-offs ≤8, and ≤ 12 have been found to correspond to depression according to the Schedules for Clinical Assessment in Neuropsychiatry and Major Depression Inventory, respectively [39, 40]. WHO-10 is suitable both as a screening tool for depression, as an outcome measure of treatment effects, and for comparison of mental well-being between population groups and over time [41]. The Swedish version of the WHO-10, used here, has shown good reliability and validity [43].

### Statistical analyses

Descriptive statistics of health and sociodemographic characteristics at T1 were obtained by calculating frequencies (*n*), proportions (%), and proportional differences using Pearson’s Chi^2^ test. To investigate potential differences in mental well-being scores between 1) non-care-seekers versus care-seekers, and 2) insufficient-care-perceivers versus sufficient care-perceivers, differences in means were calculated using independent sample t-tests, and multivariable linear regression analyses. The analyses were conducted at T1 and T2, separately. To explore if these potential differences were consistent across subgroups, t-tests were stratified by potential confounders. Multivariable linear regression analysis was used to investigate differences in mental well-being scores between the groups while controlling for potential confounders. All relevant assumptions for multivariable linear regression analyses were met [44]. Potential confounders were entered into multivariable models in steps: Model 1 included sociodemographic variables, and Model 2 added health variables. The analyses are presented as unstandardised beta-coefficients (B) with a 95% confidence interval, and R squares (R2).

Drop-out analysis was conducted by comparing characteristics of the participants lost to follow-up at T2, versus the participants, using Pearson’s Chi^2^ test and independent sample t-tests. Such analysis was also performed to compare the characteristics of those who were excluded due to missing data on the WHO-10 at T1 and/or T2, versus the final study sample. To investigate if the exclusion of these participants changed the results presented in this paper, sensitivity analyses were conducted where all analyses were performed for a sample including those 131 with missing data on WHO-10 (*n* = 1371). For all statistical analyses, the alfa level was set at *p* < 0.05. All statistical analyses were conducted using IBM SPSS Statistics, version 25.

## Results

### Loss to follow-up and analyses of missing data

Of the 1793 participants at T1, 411 were lost to follow-up at T2 (Fig. 2). These were more likely to be younger, to have secondary education (versus university education), to be born outside the Nordic countries, to have persistent mental illness, poorer mental well-being, and to have perceived a need for mental healthcare at some time in life, compared to the T2 participants (*p* < 0.05). No statistically significant differences in mental healthcare-seeking or perceived care-sufficiency were observed (see Additional file 2). The group with missing data on WHO-10 were more likely to be older, to be born outside the Nordic countries, and to have primary education (versus secondary and university education), compared to the study sample (*p* < 0.05). No statistically significant differences in persistent mental or physical illness, mental well-being, perceived need for mental healthcare, mental healthcare-seeking, or perceived care-sufficiency were observed (see Additional file 3).

### Characteristics of the study sample

Of the study sample of 1240 men, 24% (*n* = 293) reported that they had perceived a need for mental healthcare at some time in life. Of those, 37% had refrained from seeking care (*non-care-seekers,* Table 1). A higher proportion of non-care-seekers were younger, and did not have a persistent mental illness, compared to care-seekers (*p* < 0.05). Among the care-seekers, 29% reported that they had received insufficient mental healthcare when seeking it, i.e. did not receive the care that they needed (*insufficient care-perceivers,* Table 1). A higher proportion of insufficient-care-perceivers were younger, compared to sufficient-care-perceivers (*p* < 0.05).

**Table 1:** Characteristics of the sample of men^a^

		Total sample *N* = 1240	Non-need-perceivers *n* = 947 ^b^	Need-perceivers by care-seeking *n* = 293		Care-seekers by perceived care-sufficiency *n* = 182	
				Non-care-seekers	Care-seekers	Insufficient care-perceivers	Sufficient care-perceivers
				*n* = 109 ^b^ (37%)	*n* = 184^b^ (63%)	*n* = 52 ^c^ (29%)	*n* = 130 ^c^ (71%)
**Time 1, 2008**		n	% ^d^	% ^d^	% ^d^	% ^d^	% ^d^
Age, years	19–30	221	18	24	14	21	12
	31–50	567	45	51	49	56	46
	51–64	452	38	25	37	23	42
Education	Primary or less	233	19	15	20	18	21
	Secondary	568	46	57	43	41	43
	University	428	35	28	37	41	36
	Missing	11					
Birth country	Nordic	1156	94	91	91	89	92
	Others	84	6	9	9	12	8
Persistent physical illness	Yes	546	41	52	55	60	54
	No	694	59	48	45	40	46
Persistent mental illness	Yes	42	0	3	19	23	18
	No	1198	100	97	81	77	82

^a^By perceived need for mental healthcare, healthcare-seeking, and perceived sufficiency of healthcare at any time in life

^b^Stratified by “*Have you at any time felt so mentally unwell that you felt a need to seek care*?” (*No, Yes but did not seek, Yes*)

^c^Stratified by “*Do you think you received the care that you needed?*” (*No, Yes*). Disperse numbers due to *n =* 2 with missing data on the question

^d^Column proportions. Valid proportions, missing values excluded

### Comparisons of mental well-being scores using T-tests

In the total study sample, the mean mental well-being score was 18.9 at both T1 and T2 (results not shown). Non-care-seekers had mental well-being scores that were 1.7 points lower at T1, compared to care-seekers (mean 14.3 versus 16.0, *p* = 0.02, see Table 2). When stratifying these results for sociodemographic and health variables, non-care-seekers still had lower mental well-being scores in most groups at T1, although the differences were only statistically significant for a few of them (i.e. among those with university education, no persistent physical illness, and no persistent mental illness). At T2, there was no longer a difference in mental well-being scores between non-care-seekers and care-seekers (mean 15.7 versus 15.8, *p* = 0.84). In line with this, there were no consistent differences in mental well-being scores between non-care-seekers and care-seekers at T2 when stratifying these results (Table 2).

**Table 2:** Comparison of mental well-being scores between non-care-seekers versus care-seekers, and insufficient- versus sufficient care-perceivers

	By mental healthcare-seeking at any time in life						By perceived sufficiency of mental healthcare					
	Time 1, 2008			Time 2, 2009			Time 1, 2008			Time 2, 2009		
	Non-care-seekers	Care-seekers		Non-care-seekers	Care-seekers		Insufficient care-perceivers	Sufficient care-perceivers		Insufficient care-perceivers	Sufficient care-perceivers	
	Mean^a^ (*n*)	Mean^a^ (*n*)	P*	Mean^a^	Mean^a^	P*	Mean^a^ (*n*)	Mean^a^ (*n*)	P*	Mean^a^	Mean^a^	P*
Total	14.3 (*109)*	16.0 (*184)*	**0.02**	15.7	15.8	0.84	14.2 (*52)*	16.6 (*130)*	**0.02**	15.0	16.1	0.38
Age, years												
19–30	14.5 (*26)*	13.2 (*26)*	0.41	16.3	15.2	0.42	13.2 (*11)*	13.3 (*15)*	0.97	16.3	14.4	0.36
31–50	14.5 (*56)*	16.4 (*90)*	0.05	14.9	15.7	0.48	14.2 (*29)*	17.3 (*60)*	**0.03**	14.5	16.2	0.34
51–64	13.9 (*27)*	16.5 (*68)*	0.09	16.6	16.2	0.82	14.9 (*12)*	16.8 (*55)*	0.41	15.1	16.4	0.58
Education												
Primary or less	14.7 (*16)*	14.7 (*36)*	0.99	17.0	14.6	0.16	12.6 (*9)*	15.4 (*27)*	0.19	12.3	15.4	0.17
Secondary	14.6 (*60)*	15.2 (*78)*	0.55	15.8	14.9	0.43	13.6 (*21)*	15.8 (*56)*	0.19	13.3	15.5	0.20
University	13.9 (*30)*	17.4 (*68)*	**0.01**	14.9	17.5	**0.05**	15.2 (*21)*	18.3 (*46)*	0.06	17.4	17.4	0.99
Country of birth												
Nordic country	14.6 (*99)*	15.9 (*168)*	0.09	16.0	15.9	0.86	14.4 (*46)*	16.4 (*120)*	0.06	15.4	16.0	0.63
Outside Nordic	11.4 (*10)*	16.6 (*16)*	0.06	11.9	14.9	0.19	12.8 (*6)*	18.8 (*10)*	0.09	12.0	16.7	0.15
Persistent physical illness												
Yes	14.0 (*57)*	14.5 (*101)*	0.62	15.2	15.1	0.91	12.6 (*31)*	15.3 (*70)*	**0.05**	13.7	15.7	0.18
No	14.7 (*52)*	17.8 (*83)*	**0.00**	16.2	16.7	0.62	16.6 (*21)*	18.1 (*60)*	0.29	16.9	16.5	0.81
Persistent mental illness												
Yes	8.3 (*3)*	10.7 (*35)*	0.53	11.7	10.2	0.71	7.9 (*12)*	12.1 (*23)*	**0.02**	6.0	12.4	**0.01**
No	14.5 (*106)*	17.2 *(149)*	**0.00**	15.8	17.1	0.07	16.1 (*40)*	17.6 (*107)*	0.15	17.7	16.9	0.45

* *P*-value. Independent sample t-test of mean difference. Bold text indicates *p* < 0.05

^a^Lower score indicates poorer mental well-being on WHO (Ten) Well-being Index, 0–30 p

Among care-seekers, insufficient care-perceivers had mental well-being scores that were 2.4 points lower at T1, compared to sufficient-care-perceivers (mean 14.2 versus 16.6, *p =* 0.02, Table 2). When stratifying these results for sociodemographic and health variables, insufficient care-perceivers had lower mental well-being scores in all groups at T1, but the differences were only statistically significant for a few of them (i.e. among those aged 31–50 years, those with persistent physical illness, and those with persistent mental illness). At T2, there was no longer a statistically significant difference in mental well-being scores between insufficient care-perceivers and sufficient care-perceivers (mean 15.0 versus 16.1, *p* = 0.38, Table 2). When stratifying these results, insufficient care-perceivers had lower mental well-being scores in some of the groups at T2, but this was only statistically significant among those with persistent mental illness.

### Comparisons of mental well-being scores using multivariable linear regression analysis

In Table 3, we compared mental well-being scores between non-care-seekers versus care-seekers, and insufficient- versus sufficient care-perceivers using crude and multivariable linear regression. The unstandardised beta-coefficients (B) represent the differences in scores. At T1, non-care-seekers had mental well-being scores that were about two points lower in both the crude and the fully adjusted model, compared to care-seekers (Model 2, B = − 2.49, 95% CI − 3.86 to − 1.12, Table 3). However, there was no statistically significant difference in scores between non-care-seekers and care-seekers at T2 (Model 2, B = − 1.11, 95% CI − 2.59 to 0.38, Table 3). Among care-seekers, insufficient care-perceivers had mental well-being scores that were about two points lower at T1 compared to sufficient care-perceivers, in both the crude model, Model 1 (adding sociodemographic variables), and Model 2 (adding health variables). However, this difference was not statistically significant in the fully adjusted Model 2 (B = − 1.70, 95% CI − 3.53 to 0.14). There was no statistically significant difference in scores between insufficient care-perceivers and sufficient care-perceivers at T2 (Model 2, B = − 0.52, 95% CI − 2.54 to 1.50, Table 3).

**Table 3:** Comparison of mental well-being scores between non-care-seekers versus care-seekers, and insufficient- versus sufficient-care-perceivers. Crude and multivariable linear regression analyses

	Non-care-seekers vs care-seekers (among need-perceivers, ***n*** = 293)		
	Crude	Model 1^b^	Model 2^c^
Time 1, 2008			
Unstandardised B^a^	−1.65 (−3.08 to −0.22)	−1.43 (−2.88 to 0.03)	−2.49 (− 3.86 to − 1.12)
*P-*value	0.02	0.05	0.00
R2	0.02	0.04	0.20
Time 2, 2009			
Unstandardised B^a^	−0.15 (−1.68 to 1.37)	0.00 (−1.55 to 1.54)	− 1.11 (−2.59 to 0.38)
*P-*value	0.84	1.00	0.14
R2	0.00	0.02	0.15
	**Insufficient vs sufficient care-perceivers (among care-seekers,** ***n =*** **182)**		
Time 1, 2008			
Unstandardised B^a^	−2.44 (−4.47 to −0.41)	− 2.29 (− 4.35 to − 0.23)	−1.70 (−3.53 to 0.14)
*P-*value	0.02	0.03	0.07
R2	0.03	0.08	0.28
Time 2, 2009			
Unstandardised B^a^	−1.07 (−3.24 to 1.10)	−1.02 (− 3.23 to 1.18)	−0.52 (−2.54 to 1.50)
*P-*value	0.33	0.36	0.61
R2	0.01	0.04	0.21

^a^Represents the difference in scores. Negative values indicate lower mental well-being scores on WHO (Ten) Well-being Index, 0–30 p. 95% confidence intervals

^b^Adjusted for age category, education, country of birth

^c^Adjusted for age category, education, country of birth, persistent physical illness, and persistent mental illness

**Fig. 3:**
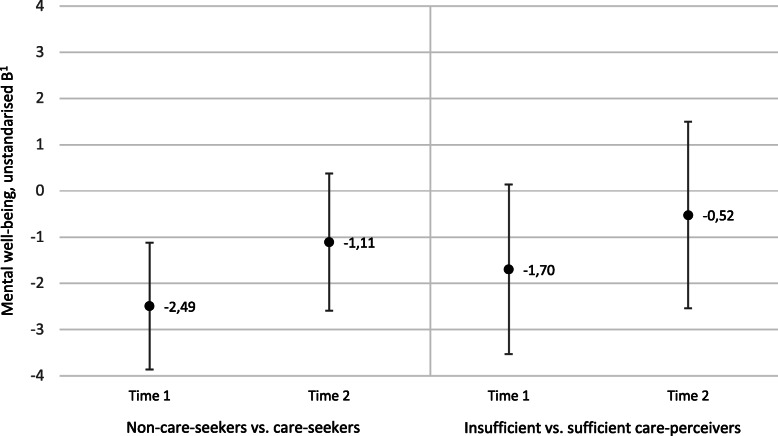
Comparison of mental well-being scores between non-care-seekers versus care-seekers, and insufficient- versus sufficient care-perceivers, at Time 1 (2008) and Time 2 (2009). ^1^ Represents the difference in mental well-being scores. Negative values indicate lower mental well-being scores on WHO (Ten) Well-being Index, 0–30 p. Unstandardised B with 95% confidence intervals based on fully adjusted multivariable linear regression analyses

In sum, non-care-seekers were more likely to have lower mean mental well-being scores at T1, but not at T2, also when controlling for potential confounders. Insufficient care-perceivers were not more likely to have lower mean mental well-being scores at T1 or T2 when adjusting for potential confounders. The main results, based on the fully adjusted linear regression analyses, are illustrated in Fig. 3. Figure 3 shows that there was a tendency for lower mean mental well-being scores among non-care-seekers also at T2, and among insufficient care-perceivers at both T1 and T2, although the results were not statistically significant, as shown by the confidence intervals.

### Sensitivity analysis

The sensitivity analyses, including those that had missing data on the WHO-10 at T1 and/or T2, were consistent with the results presented above, with one exception. The multivariable linear regression analyses showed a statistically significant association between being an insufficient care-perceiver and having lower mental well-being scores at T1, also in the fully adjusted model adding health variables, in contrast to the results above (Model 2, B = -1.91, 95% CI − 3.71 to − 0.10, see Additional file 4).

## Discussion

This is the first longitudinal study on a population-based sample of men in Sweden investigating the hypotheses that men who have previously refrained from seeking mental healthcare, or perceived the care as insufficient when seeking it, have poorer mental well-being than men who sought care and perceived it as sufficient (see Fig. 1). We observed that 37% of the men who had perceived a need for mental healthcare at some time in life had refrained from seeking it. Among those who had sought care, 29% perceived that they had received insufficient care. Our hypotheses were only partially supported: We found 1) poorer mental well-being among non-care-seekers at T1, 2) an indication of poorer mental well-being among insufficient-care-perceivers at T1, but 3) no statistically significant differences at T2. Therefore, hypothesis A1 was confirmed, there was some support for hypothesis B1, but hypotheses A2 and B2 were rejected (see Fig. 1).

### Poorer mental well-being among non-care-seekers at T1

The observed poorer mental well-being among non-care-seekers at T1 is worrying, as poor mental well-being using WHO-10 is associated with a higher likelihood for depression [39–41] and suicidality [41]. This result is in line with the large body of research showing the benefits of receiving treatment [4, 5]. More importantly, it supports the suggestion that refraining from seeking mental healthcare is detrimental for men’s mental well-being also on a population level [7–11]. The poorer mental well-being may reflect a risk for more severe consequences, such as premature death and suicide, and the use of maladaptive coping strategies, such as high alcohol consumption [7], previously explained by adherence to health-hazardous masculinity norms [21]. However, more research is needed to confirm this result and investigate why non-care-seekers had poorer mental well-being. Is it the lack of treatment in itself or other factors? For example, non-care-seekers may have refrained from seeking care due to stigma and embarrassment [26, 45, 46], which in itself is associated with poorer quality of life [47], social isolation, and drinking to cope [48].

It should be noted that the difference in mental well-being scores between non-care-seekers and care-seekers at T1 was small. However, even small differences may have major implications on population level given the high prevalence of depression, anxiety- and alcohol use disorders [1], and refraining from seeking mental healthcare among men [6, 26]. In addition, this study only investigated differences in mental well-being among *need-perceivers*, as the measure of care-seeking was self-reported (i.e., “Have you at any time felt so mentally unwell *that you felt a need to seek care?*”). However, a large proportion of men with depression do not perceive a need for care [26, 49]. Potentially, this group may suffer from even poorer outcomes. Therefore, this study may have underestimated the detrimental outcomes of not seeking care.

### Indication of poorer mental well-being among insufficient care-perceivers at T1

Even in insufficient care-perceivers, the t-tests and the linear regression analysis showed poorer mental well-being at T1, compared to sufficient care-perceivers. However, this result was not statistically significant when controlling for sociodemographic and health variables (Table 3). This is probably due to the small sample in this sub-group analysis, as the sensitivity analysis on a larger sample showed a statistically significant difference (see Additional file 4). Our result is in line with previous research that has shown an association between dissatisfaction with care and depression [37, 38]. The indicated poorer mental well-being among insufficient care-perceivers may be due to not receiving care of adequate quality [20], as consistent evidence shows a positive association between perceived sufficiency of care and quality of care [27].

However, as we had no measure of clinical sufficiency of the care, it is also possible that insufficient care-perceivers were offered care of adequate standards but did not find it appropriate based on their perceived needs. The perception of need for care is a complex process, impacted by e.g., expectations of care, knowledge about mental illnesses, cultural and religious beliefs, stigmatizing attitudes, and gender norms [50, 51]. For example, traditional masculinity norms can make it difficult to identify with a person needing mental health treatment, with a negative impact on men’s adherence and treatment efficacy [21]. Masculinity norms can also increase men’s self-stigma, and make men more sceptical and non-adherent to treatment [21]. These barriers may negatively impact the perceived sufficiency of the care. The healthcare system should help men to overcome these barriers by providing high-quality mental healthcare adapted to men’s needs. Therefore, more knowledge is needed on what kind of mental healthcare different groups of men find appropriate and sufficient based on their needs.

### Lack of differences in mental well-being at T2

Although the expected differences in mental well-being between non-care-seekers and care-seekers, and insufficient care-perceivers and sufficient care-perceivers, were observed at T1, we found no statistically significant differences 1 year later, at T2. As there was a tendency towards poorer mental well-being at T2 (Fig. 3), the lack of statistically significant differences could reflect a limited statistical power in these analyses. The lack of differences could also reflect remission of symptoms, as previous studies show that the majority of those with common mental disorders who do not seek treatment remit [36, 52, 53]. Results from a longitudinal study on men and women showed that among persons with untreated depression, anxiety, or substance disorder, 50% remitted within 3 years [52]. A Swedish study also showed that the most common reason for refraining from seeking mental healthcare was believing that the condition would resolve by itself [26]. However, our results do not support any complete remission of symptoms, as both non-care-seekers and care-seekers (regardless of the perceived sufficiency of the care) still had mean mental well-being scores below the population mean at T2 (15.7, and 15.8, compared to the population mean 18.9). This is worrying, as the population mean should be the goal for complete remission [41]. The lack of full recovery at T2 highlights a need for improved mental healthcare, but also a need to target societal factors outside healthcare that may have greater importance for men’s mental well-being.

It should be noted that we lack data on care-seeking between T1 and T2, which could explain the improved mental well-being of non-care-seekers and insufficient care-perceivers. They may have sought and received sufficient care between T1 and T2. We also lack data on informal help-seeking among family and friends and access to other resources (e.g., social, psychological, and economic). Privileged groups with milder conditions may improve their mental well-being using self-help strategies. Other groups of men may be more vulnerable to severe consequences [54], due to social position and/or a greater clinical need for care. This is indicated by our stratified analyses (Table 2). Among those with persistent mental illness, insufficient care-perceivers had poorer mental well-being at both T1 and T2. Among those born *outside* Nordic countries, non-care-seekers and insufficient care-perceivers had poorer mental well-being than the corresponding groups born *in* Nordic countries, at both T1 and T2, although the results were not statistically significant. These results show the value of using both measurement points for mental well-being. However, these results should be treated with great caution due to the small numbers in the sub-samples. Future studies with larger samples are needed, that allow for stratified analyses of differences among groups of men.

In sum, this study gives some support to the hypotheses that men who have previously refrained from seeking mental healthcare or perceived the care as insufficient when seeking it have poorer mental well-being than men who have sought care and perceived it as sufficient. However, the relatively small differences at T1 and the lack of persistent differences at T2 point in another direction.

### Methodological considerations

The major limitation of this study is the use of secondary data. One drawback is that the questions measuring the exposures (i.e., refraining from seeking care, or perceiving the care as insufficient) referred to “any time in life” but were measured at T1. Therefore, potential outcomes, e.g., poorer mental well-being, could have occurred before T1, at T1, and/or at T2. Due to this life-time measure, and the observational nature of the data, any conclusions about causality should be drawn with great caution. For future studies, we recommend using a more specified time frame for the exposures, e.g., care-seeking within the last 12 months. However, this would require much larger samples to be able to conduct statistical analyses.

The rationale for still using the life-time measure is the lack of previous research within the field. Also, we believe that the participants replied to the questions on care-seeking based on what was their most recent or severe experience, as refraining from seeking care, or perceiving the care as insufficient is not necessarily a binary event. This could explain why we found differences in mental well-being at T1 but not at T2, as T2 may have been too far from the event to observe an effect.

However, the longitudinal design, with measurement of mental well-being at both T1 and T2, has several advantages. Firstly, a difference between groups observed at two time points is more reliable than a difference observed at one time point only, and could imply a more stable effect. Secondly, the results from T2 allowed us to challenge the cross-sectional results from T1. For example, the lack of persistent differences at T2 highlights a potential uncertainty of the findings at T1. Thirdly, the risk for reverse causality decreased at T2. At T1, those with poorer mental well-being may be more likely to report that they had previously received insufficient care, due to current pessimism related to depression [55, 56]. Therefore, reporting insufficient care may have been an outcome rather than an exposure. This may explain why we found poorer mental well-being among insufficient care-perceivers at T1, but not at T2. Future studies should combine subjective measures as perceived sufficiency of the care with more objective measures, e.g., register data on receiving care of adequate standard based on evidence-based guidelines.

Another aspect to consider is the participation rate of 34%. It may be problematic if participation was selective, e.g. if the association between the exposures and the outcome was stronger among non-participants. There is some research pointing in this direction. Non-participating men have been shown to be less likely to seek care [57], and non-participants have been shown to be more likely to have a psychiatric disorder than participants [58]. This is in line with our finding that those lost to follow-up were more likely to have poor mental well-being and persistent mental illness. However, we found no differences in care-seeking. Plausibly, non-participants share characteristics related to the study’s exposures and outcomes, e.g. belonging to groups in adverse life situations who would benefit the most from treatment. Therefore, this study could have underestimated the negative effect of not receiving care. Also, the relatively low participation rate, and the skewed participation based on sociodemographic characteristics [34] may have contributed to the limited statistical power in the adjusted and stratified analyses, leading to a risk of undetected true differences, i.e. a type II error.

Also, it should be mentioned that the data is relatively old. Men’s mental healthcare-seeking and masculinity norms have received increasing attention in Sweden since the data was collected in 2008 and 2009 [59]. Although behaviours and norms are relatively stable over time, societal changes may impact the results. New research is needed, especially in the light of the corona pandemic with its large impact on mental health and healthcare systems.

In sum, due to these limitations the result should be generalised with caution. Future research is needed using primary data and more refined methodology, including a more specific time frame for the assessment of the exposures, longer follow-up, and larger sample sizes. However, this study also has some relevant strengths, namely: 1) the relatively large population-based sample of men, 2) inclusion of both refraining from seeking care *and* perceiving the care as insufficient as exposures 3), access to longitudinal data on mental well-being, 4) the use of the reliable and validated instrument WHO-10 [42, 43], and 5) stratified data on sociodemographic and health characteristics. In addition, this is one of the very few longitudinal population-based studies investigating if men with prior unmet need for mental healthcare have poorer mental well-being, and the only such study from Sweden. Therefore, we believe that this study adds to the literature on men’s mental healthcare-seeking.

### Implications

The results of this study are inconclusive and have to be interpreted with the study’s limitations in mind. The poorer mental well-being among non-care-seekers and insufficient care-perceivers at T1, and the lack of statistically significant differences at T2, has to be confirmed in larger studies with more refined methodology. Meanwhile, the indication of poorer mental well-being among non-care-seekers and insufficient care-perceivers at T1 suggests that men’s unmet need for mental healthcare is a significant public health problem.

However, the relatively small differences in mental well-being may reflect that mental healthcare only represents a part of what is important for men’s mental well-being. Therefore, efforts to increase men’s mental well-being have to be conducted not only within the healthcare system but also on a societal level. One way to go forward is to target masculinity norms [59], that impact not only men’s mental healthcare-seeking and perceived sufficiency of the care [7], but men’s health-hazardous behaviours in general.

## Conclusion

This is the first longitudinal study on a population-based sample of men in Sweden investigating if men who have previously refrained from seeking mental healthcare, or perceived care as insufficient when seeking it, have poorer mental well-being than those that have not. The results show that these men have poorer mental well-being at the first time point, but the difference is small, and there is no difference 1 year later. However, due to the lack of data on the exact time of these exposures, further longitudinal studies are needed, using a more refined methodology. In addition, we suggest interventions to increase men’s mental healthcare-seeking and to provide mental healthcare that is perceived as sufficient. This should be done both in the healthcare system and on a societal level by targeting health-hazardous masculinity norms that influence men’s risk behaviours and perceptions about mental healthcare.

## Supplementary Information

**Additional file 1.** Directed Acyclic Graphs. Directed acyclic graphs showing potential confounders, moderators and mediators for the relationship between the exposures and the outcome.

**Additional file 2.** Supplementary table. Characteristics of those lost to follow-up at Time 2. Characteristics of those lost to follow-up at Time 2.

**Additional file 3.** Supplementary table. Characteristics of those with missing data on WHO (Ten) Well-being Index (WHO-10). Description of data: Characteristics of those with missing data on WHO (Ten) Well-being Index.

**Additional file 4.** Supplementary table. Sensitivity analysis. Sensitivity analysis including those that had missing data on WHO (Ten) Well-being Index at T1 or T2. Comparison of mental well-being scores, using crude and multivariable linear regression.

## Data Availability

The data that support the findings of this study are available from the Swedish National Data Service, https://snd.gu.se/sv/catalogue/study/snd0870, but restrictions apply to the availability of this data.

## Abbreviations

T1: Time 1, year 2008
T2: Time 2, year 2009
HAP: Health Assets Project
WHO-10: WHO (Ten) Well-being Index
B: Unstandardised beta-coefficients
R2: R squares
