# Supplementary figures and images for: Poorer mental well-being and prior unmet need for mental healthcare: a longitudinal population-based study on men in Sweden
Source: Arch Public Health. 2021 Nov 3;79:189. doi: 10.1186/s13690-021-00706-0 (PMC8564598; doi:10.1186/s13690-021-00706-0)

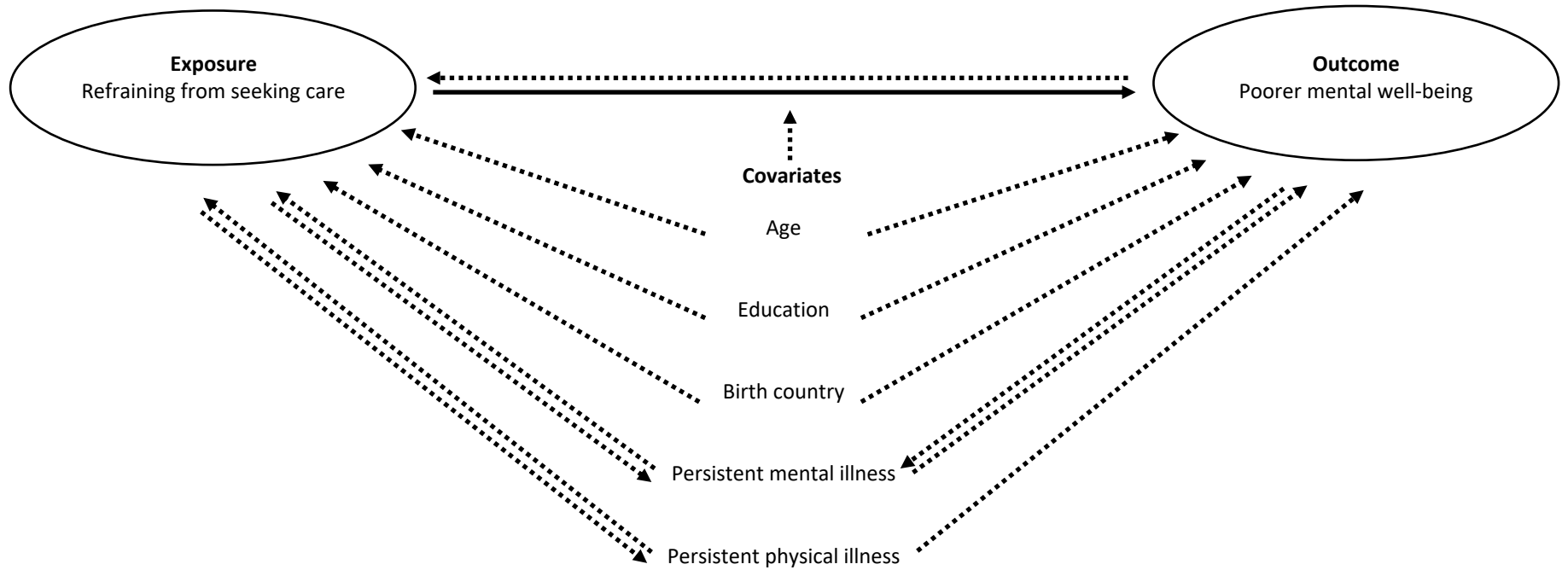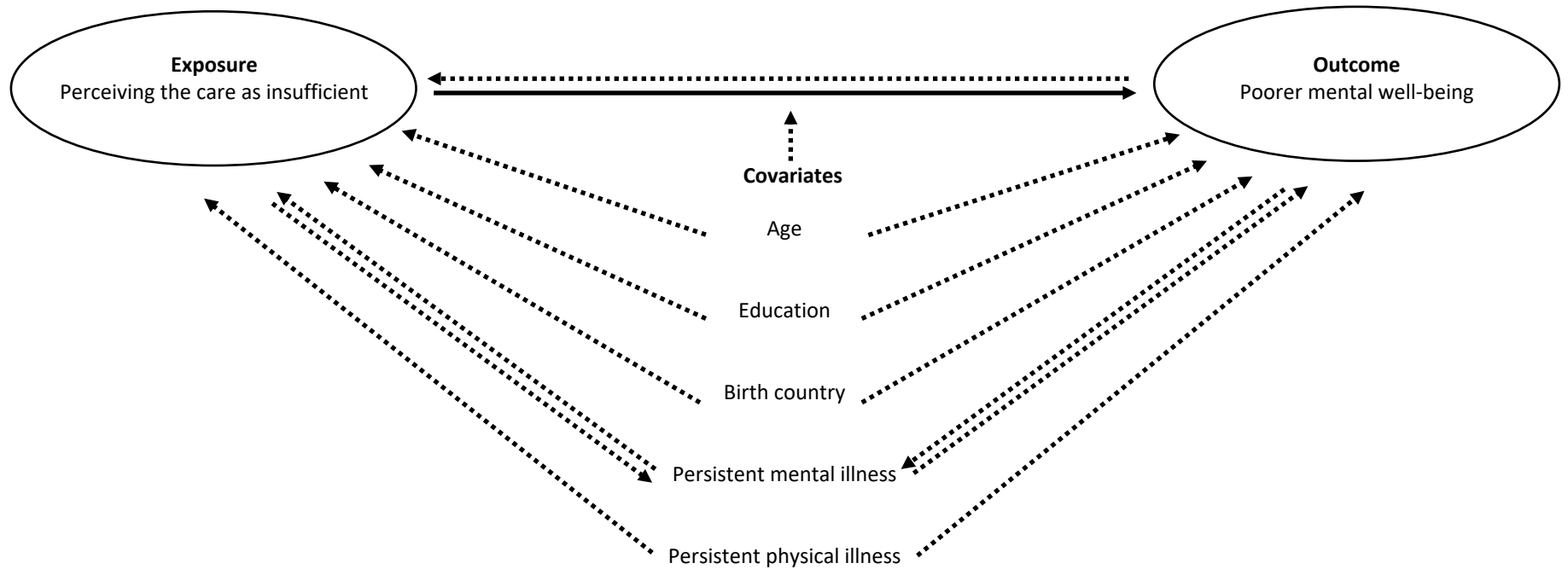

Supplement: Supplementary file 1 — Additional file 1. Directed Acyclic Graphs. Directed acyclic graphs showing potential confounders, moderators and mediators for the relationship between the exposures and the outcome. [file 13690_2021_706_MOESM1_ESM.pdf]
