# Supplementary material for: Poorer mental well-being and prior unmet need for mental healthcare: a longitudinal population-based study on men in Sweden
Source: Arch Public Health. 2021 Nov 3;79:189. doi: 10.1186/s13690-021-00706-0 (PMC8564598; doi:10.1186/s13690-021-00706-0)
Supplement: Supplementary file 2 — Additional file 2. Supplementary table. Characteristics of those lost to follow-up at Time 2. Characteristics of those lost to follow-up at Time 2. [file 13690_2021_706_MOESM2_ESM.docx]

| Additional file 2. Supplementary table. Characteristics of those lost to follow-up at time 2. | | | |
| --- | --- | --- | --- |
|  |  | Lost to follow-up at Time 2 | Participants  at  Time 2 |
|  |  | n=411  (23%) | n=1382  (77%) |
|  |  |  |  |
| **Time 1, 2008** |  | % ^a^ | % ^a^ |
| Age, years | 19-30 | 32 | 17 |
|  | 31-50 | 44 | 45 |
|  | 51-64 | 24 | 38 |
| Education | Primary or less | 19 | 20 |
|  | Secondary | 53 | 46 |
|  | University | 28 | 34 |
| Birth country | Nordic | 83 | 91 |
|  | Others | 17 | 9 |
| Persistent physical illness | Yes | 42 | 45 |
|  | No | 58 | 55 |
| Persistent mental illness | Yes | 6 | 3 |
|  | No | 94 | 97 |
|  |  |  |  |
| Perceived need for mental healthcare | Need-perceivers | 31 | 24 |
|  | Non-need-perceivers | 69 | 76 |
| Healthcare-seeking ^b^ | Non-care-seekers | 32 | 37 |
|  | Care-seeker | 68 | 63 |
| Perceived sufficiency of healthcare ^c^ | Insufficient care-perceivers | 31 | 29 |
|  | Sufficient care-perceivers | 69 | 71 |
| Mental well-being score^d^ | Mean | 18.0 | 18.9 |

^a^ Column proportions. Valid proportions, missing values excluded

^b^ Subgroup analysis among need-perceivers

^c^ Subgroup analysis among care-seekers

^d^ Lower score indicates poorer mental well-being on WHO (Ten) Well-being Index, 0-30 p.
