# Supplementary material for: Poorer mental well-being and prior unmet need for mental healthcare: a longitudinal population-based study on men in Sweden
Source: Arch Public Health. 2021 Nov 3;79:189. doi: 10.1186/s13690-021-00706-0 (PMC8564598; doi:10.1186/s13690-021-00706-0)
Supplement: Supplementary file 3 — Additional file 3. Supplementary table. Characteristics of those with missing data on WHO (Ten) Well-being Index (WHO-10). Description of data: Characteristics of those with missing data on WHO (Ten) Well-being Index. [file 13690_2021_706_MOESM3_ESM.docx]

| Additional file 3. Supplementary table. Characteristics of those with missing data on WHO (Ten) Well-being Index (WHO-10). | | | |
| --- | --- | --- | --- |
|  |  | Missing data on WHO-10 at Time 1 and/or Time 2 | Study sample |
|  |  | n=131  (10%) | n=1240  (90%) |
|  |  |  |  |
| **Time 1, 2008** |  | % ^a^ | % ^a^ |
| Age, years | 19-30 | 10 | 18 |
|  | 31-50 | 37 | 46 |
|  | 51-64 | 53 | 37 |
| Education | Primary or less | 29 | 19 |
|  | Secondary | 41 | 46 |
|  | University | 30 | 35 |
| Birth country | Nordic | 74 | 93 |
|  | Others | 26 | 7 |
| Persistent physical illness | Yes | 52 | 44 |
|  | No | 48 | 56 |
| Persistent mental illness | Yes | 4 | 3 |
|  | No | 96 | 97 |
|  |  |  |  |
| Perceived need for mental healthcare | Need-perceivers | 27 | 24 |
|  | Non-need-perceivers | 73 | 76 |
| Healthcare-seeking ^b^ | Non-care-seekers | 39 | 37 |
|  | Care-seekers | 61 | 63 |
| Perceived sufficiency of healthcare ^c^ | Insufficient care-perceivers | 30 | 29 |
|  | Sufficient care-perceivers | 70 | 71 |
| Mental well-being score^d^ | Mean | 19.2 | 18.9 |

^a^ Column proportions. Valid proportions, missing values excluded

^b^ Subgroup analysis among need-perceivers

^c^ Subgroup analysis among care-seekers

^d^ Lower score indicates poorer mental well-being on WHO (Ten) Well-being Index, 0-30 p.
