# Supplementary material for: Poorer mental well-being and prior unmet need for mental healthcare: a longitudinal population-based study on men in Sweden
Source: Arch Public Health. 2021 Nov 3;79:189. doi: 10.1186/s13690-021-00706-0 (PMC8564598; doi:10.1186/s13690-021-00706-0)
Supplement: Supplementary file 4 — Additional file 4. Supplementary table. Sensitivity analysis. Sensitivity analysis including those that had missing data on WHO (Ten) Well-being Index at T1 or T2. Comparison of mental well-being scores, using crude and multivariable linear regression. [file 13690_2021_706_MOESM4_ESM.docx]

| Additional file 4. Supplementary table. Sensitivity analysis. Comparison of mental well-being scores between non-care-seekers versus care-seekers, and insufficient- versus sufficient-care-perceivers. Crude and multivariable linear regression analyses ^a^. | | | |
| --- | --- | --- | --- |
|  | **Non-care-seekers vs care-seekers (among need-perceivers, n=329)** | | |
|  | Crude | Model 1^2^ | Model 2^3^ |
| Time 1, 2008 |  |  |  |
| Unstandardised B^1^ | -1.66 (-3.06 to -0.26) | -1.38 (-2.80 to 0.05) | -2.45 (-3.81 to -1.10) |
| P-value | 0.02 | 0.06 | 0.00 |
| R2 | 0.02 | 0.04 | 0.19 |
| Time 2, 2009 |  |  |  |
| Unstandardised B^1^ | -0.20 (-1.66 to 1.25) | 0.03 (-1.45 to 1.52) | -1.08 (-2.51 to 0.34) |
| P-value | 0.78 | 0.97 | 0.14 |
| R2 | 0.00 | 0.03 | 0.16 |
|  | | | |
|  | **Insufficient vs sufficient care-perceivers (among care-seekers, n=202)** | | |
| Time 1, 2008 |  |  |  |
| Unstandardised B^1^ | -2.55 (-4.54 to -0.57) | -2.52 (-4.35 to -0.50) | -1.91 (-3.71 to -0.10) |
| P-value | 0.01 | 0.01 | 0.04 |
| R2 | 0.03 | 0.08 | 0.28 |
| Time 2, 2009 |  |  |  |
| Unstandardised B^1^ | -0.75 (-2.86 to 1.36) | -0.72 (-2.86 to 1.41) | -0.16 (-2.10 to 1.79) |
| P-value | 0.48 | 0.51 | 0.87 |
| R2 | 0.00 | 0.05 | 0.23 |

^a^ Including those that had missing data on WHO (Ten) Well-being Index at T1 or T2.

^1^ Represents the difference in scores. Negative values indicate lower mental well-being score on WHO (Ten) Well-being Index, 0-30 p. 95% confidence intervals.

^2^ Adjusted for age category, education, country of birth.

^3^ Adjusted for age category, education, country of birth, persistent physical illness, and persistent mental illness.
